# A Coin-Like Peripheral Small Cell Lung Carcinoma Associated with Acute Paraneoplastic Axonal Guillain-Barre-Like Syndrome

**DOI:** 10.1097/MD.0000000000000910

**Published:** 2015-06-05

**Authors:** Ioan Jung, Simona Gurzu, Rodica Balasa, Anca Motataianu, Anca Otilia Contac, Ioana Halmaciu, Septimiu Popescu, Iunius Simu

**Affiliations:** From the Department of Pathology (IJ, SG, AOC, SP); Department of Neurology (RB, AM); and Department of Radiology, University of Medicine and Pharmacy of Tirgu-Mures, Tirgu-Mures, Romania (IH, IS).

## Abstract

A 65-year-old previously healthy male heavy smoker was hospitalized with a 2-week history of progressive muscle weakness in the lower and upper extremities. After 10 days of hospitalization, urinary sphincter incompetence and fecal incontinence were added and tetraparesis was established. The computer-tomography scan examination revealed a massive right hydrothorax and multifocal solid acinar structures with peripheral localization in the left lung, which suggested pulmonary cancer. Bone marrow metastases were also suspected. Based on the examination results, the final diagnosis was acute paraneoplastic axonal Guillain-Barre-like syndrome. The patient died 3 weeks after hospitalization. At autopsy, bronchopneumonia and a right hydrothorax were confirmed. Several 4 to 5-mm-sized round peripherally located white nodules were identified in the left lung, without any central tumor mass. Under microscope, a coin-shaped peripheral/subpleural small cell carcinoma was diagnosed, with generalized bone metastases. A huge thrombus in the abdominal aorta and acute pancreatitis was also seen at autopsy. This case highlights the difficulty of diagnosis of lung carcinomas and the necessity of a complex differential diagnosis of severe progressive ascending neuropathies. This is the 6th reported case of small cell lung cancer-associated acute Guillain-Barre-like syndrome and the first report about an association with a coin-like peripheral pattern.

## INTRODUCTION

Lung cancer is the most common tumor that occurs in heavy smokers, with squamous cell carcinoma and small cell lung carcinoma (SCLC) being the 2 most diagnosed histologic types. In patients with SCLC, the tumor can be diagnosed in 10% of cases based on an extremely wide variety of paraneoplastic syndromes.^1^ Except for the well-known syndromes that can mimic Cushing disease or Addisons syndrome, and also diffuse skin hyperpigmentation, other strange lesions have been identified. For example, localized hyperpigmentation in palms that resolved after chemotherapy, myelodysplastic syndrome, coagulopathy (Trousseaus syndrome), hypoglycemia, hyponatremia, encephalomyelopathy, cerebellar degeneration, opsoclonus myoclonus ataxia, and Eaton-Lambert myastenic syndrome were the first clinical signs reported in lung carcinomas.^2–5^ However, in very few cases, more than 2 paraneoplastic phenomena were reported to be associated.

In this article, we present the first case of a diffuse “coin-like” SCLC that was diagnosed based on an acute course of paraneoplastic axonal form of Guillain-Barre-like syndrome. To the best of our knowledge, another 5 cases with similar clinical evolution to SCLC have been published previously,^5–9^ but none reporting on a “coin-like” pattern. Further, none of the previously reported cases presented associated hypercoagulability, pancytopenia, and/or Cushing-like symptoms, such as in this case. In addition to the case presentation, a review of the possible mechanism and differential diagnosis of Guillain-Barre-like syndrome was done.

## CASE PRESENTATION

A 65-year-old previously healthy male heavy smoker and alcoholic presented in a tertiary medical center with a 2-week history of muscle weakness in the inferior limbs and ankle edema. Because he also presented coughing and fever, large spectrum antibiotics were prescribed for bronchopneumonia, alcoholic neuropathy was also suspected. During the 10-day hospitalization period, the patient's status worsened and the muscle weakness also involved the upper extremities. Steroids were given without any benefits.

The patient was transferred to our university medical center for supplementary investigations. The first physical exam revealed dysmetria and bilateral abolition of osteotendinous reflexes. His blood pressure was 120/60 mm Hg. During hospitalization at the Neurology Department, peripheral tetraparesis with generalized amyotrophy was established, urinary and fecal incontinence was associated after 3 days. Table 1 presents the patient's serum parameters; he did not consent for lumbar puncture.

**TABLE 1:**
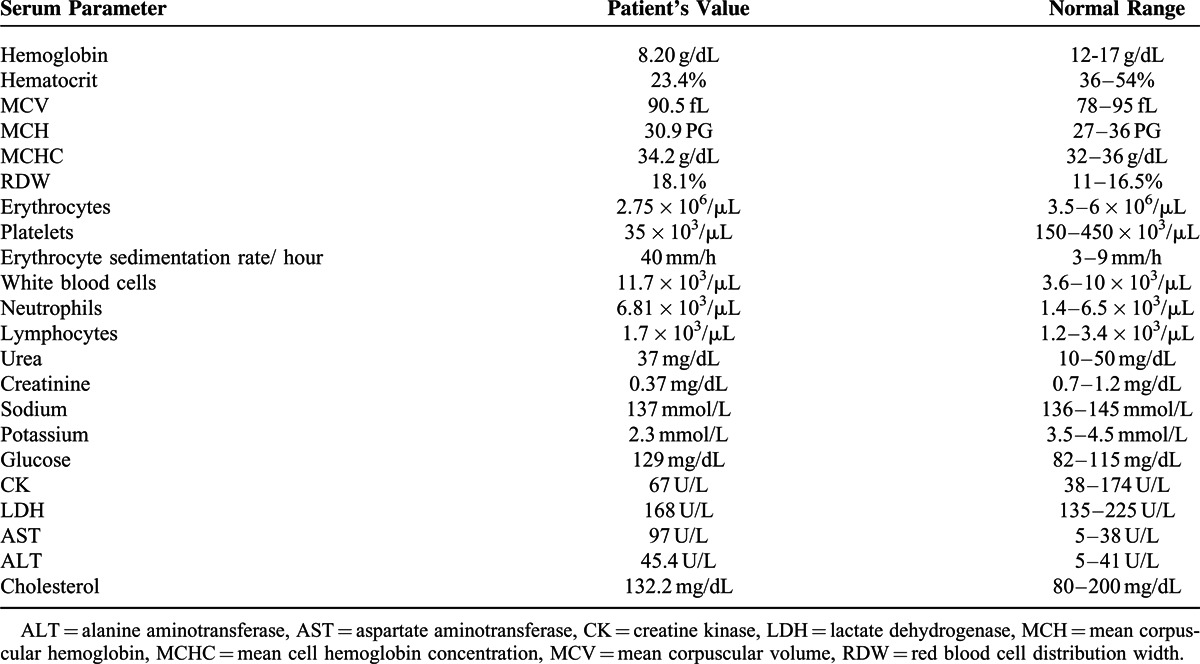
Serum parameters of the patient

**FIGURE 1:**
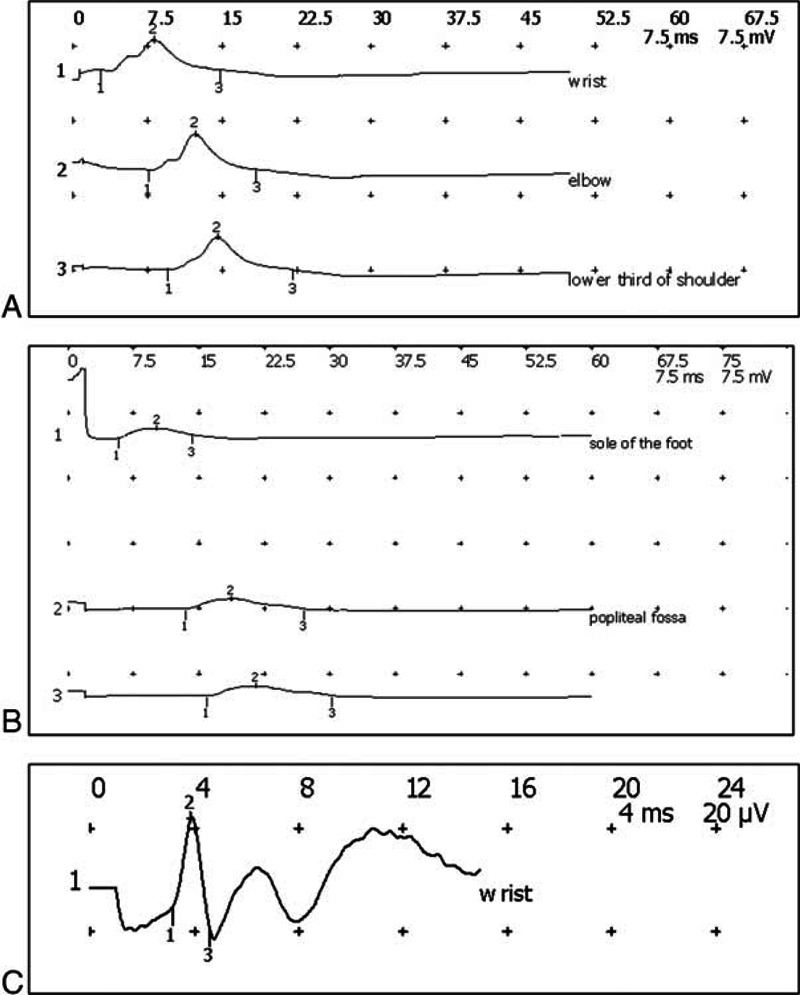
Electromyographic examination. (A) Left ulnar motor study, recording on abductor digiti minimi, C8 T1; (B) right peroneus motor study, recording on Extensor digitorum brevis; (C) right ulnaris sensory study.

At the electromyography examination, nerve conduction studies show completely normal sensory studies in upper and lower extremities. The motor studies were consistent with axonal loss (more severe in lower limbs): lLow amplitude, and slightly decreased velocities and altencies. The median and ulnar F responses were slightly prolonged. On needle electromyography there was evidence of distal denervation in the legs and arms, with fibrillation potentials and large, long, polyphasic motor unit action potentials with reduced recruitment. Comparing 2 controlateral muscles, the tibialis anterior and extensor digitorum, the findings were symmetric. There was electrophysiologic evidence of an active pure motor distal axonal polyneuropathy (Figure 1 and Table 2).

**TABLE 2:**
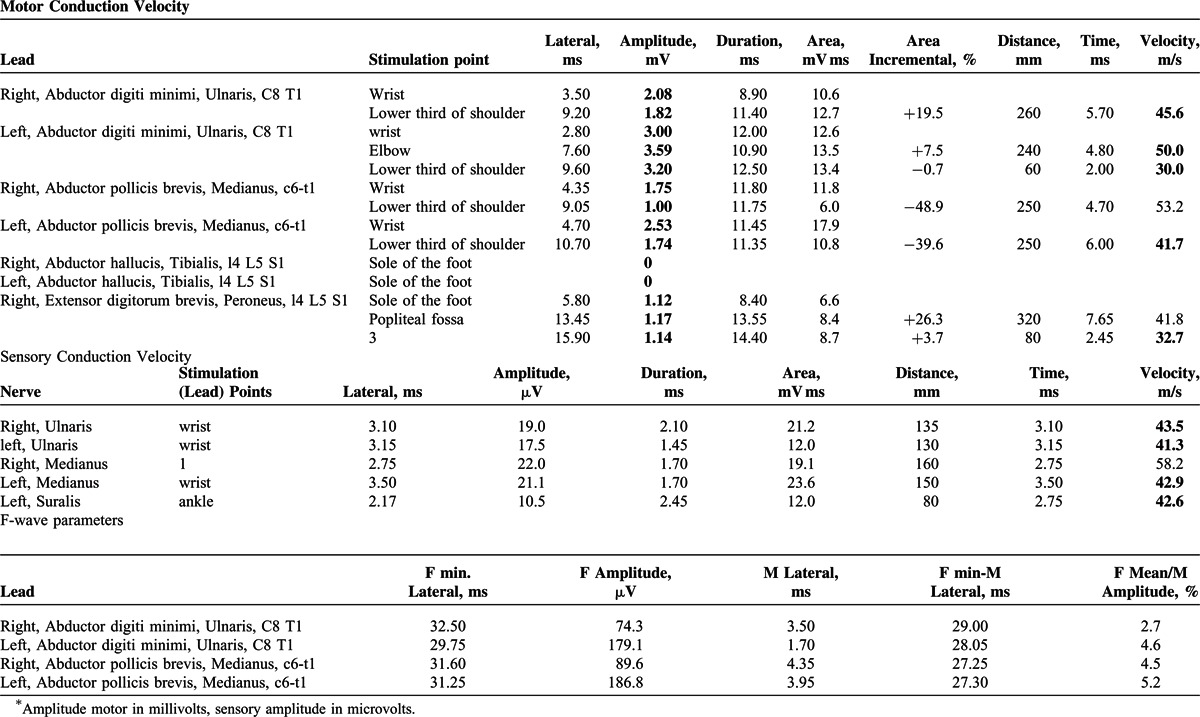
Nerve Conduction Studies

Due to the patient's history of heavy smoking—1 pack of cigarettes daily for the last 40 years—the Cushingoid (“moon-like”) aspect of the face and associated shortness of breath and coughing, paraneoplastic neuropathy was suspected. The serum level of cortisol, adrenocorticotropic hormone, or any other hormone proteins was not determined.

**FIGURE 2:**
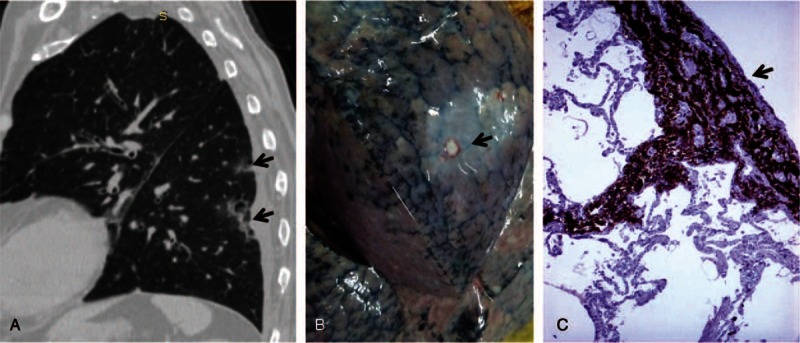
In a patient with lung cancer, the chest CT scan (lung window) revealed multifocal peripheral subpleural acinar cavitating lesions (A), which at autopsy were seen as white nodules with pleural involvement (B). Under microscope, the tumor cells presented a coin-like pattern and displayed CD56 positivity (C). CD = cluster of differentiation, CT = computer-tomography.

**FIGURE 3:**
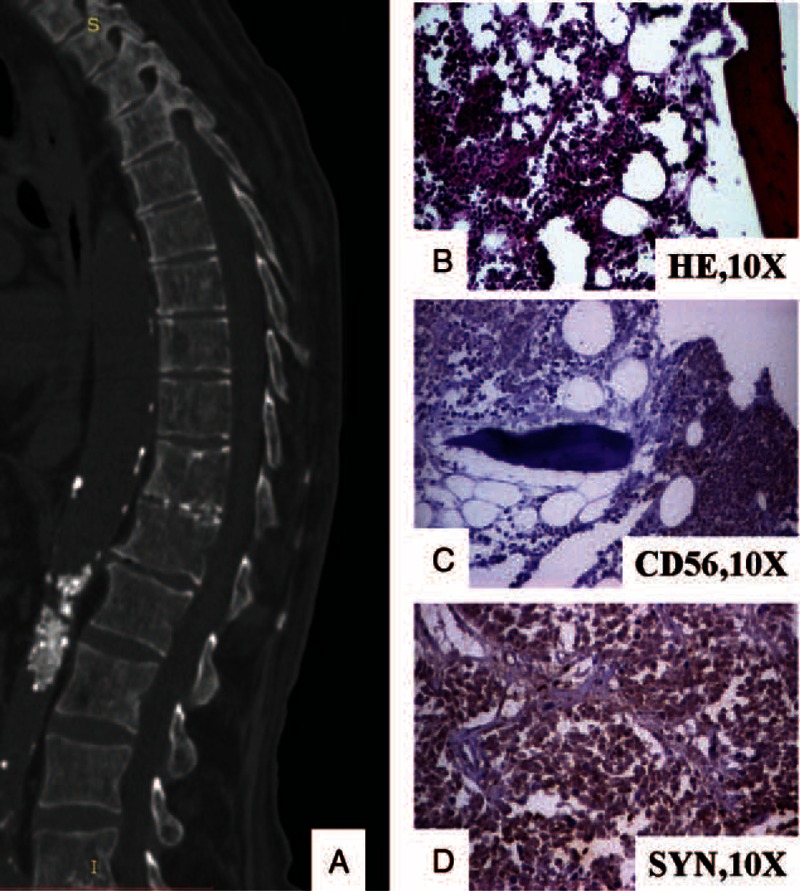
The vertebral metastases are seen as multiple osteoblastic-osteolytic foci at CT-scan (A). Under microscope, small cells can be observed in the bone marrow (B) that are marked by CD56 (C) and synaptophysin (D). CD = cluster of differentiation, CT = computer-tomography.

X-ray and computer-tomography (CT) scan examinations (lung and mediastinal windows) revealed an atelectatic area of condensation in the inferior lobe of the right lung, with fluid collection in the right pleural cavity. In the left lung, ill-regulated multifocal subpleural cavitating lesions of acinar aspect were noted, which were considered as tumor nodules (Figure 2). Mixed osteoblastic-osteolytic lesions were observed in thoraco-lumbar vertebrae, which corresponded to bone marrow metastases (Figure 3); a calcified area was also seen in the lumen of the abdominal aorta. The patient was scheduled for transfer to the Oncology Department, but he died in our clinic after another 2 days of hospitalization.

At autopsy, the macroscopic examination confirmed bronchopneumonia and unilateral right hydrothorax (500 g transudate), without tumor nodules in the right lung. Hydropericardium was associated (400 g). Examination of the left lung parenchyma indicated bronchopneumonia, without any central tumor mass. A particular aspect of the case was the presence of multiple 4 to 5-mm-sized white elastic peripheral nodules with pleural involvement (Figure 2), which were fixed in formalin and embedded in paraffin for further microscopic examination. Iliac crest bone grafting was also done for microscopic investigation. Except for moderate edema, no other brain lesions were identified. The adrenal glands presented a normal structure. A huge pseudo-obstructive thrombus was seen in the abdominal aorta; acute pancreatitis with large hemorrhagic necroses and liver steatosis was associated.

Microscopic examination of the peripheral pulmonary masses showed multiple “coin-shaped” tumor nodules with small round cells marked by the neuroendocrine markers cluster of differentiation (CD)56 and synaptophysin (Figure 2). Cords and nests of small round tumor cells, marked by CD56 and synaptophysin, were also seen in bone marrow from the iliac crest bone (Figure 3). The vertebral column was not examined under microscope.

Based on the macroscopic and microscopic features and clinical picture, the final diagnosis was “Coin-like peripheral SCLC with bone metastases and paraneoplastic axonal form of Guillain-Barre-like syndrome.” The hypercoagulability could be a paraneoplastic syndrome or could be related to the pancreatitis. Being an autopsy case, the ethical approval or patient consent was not necessary.

## DISCUSSION

Differential diagnoses between primary neurologic disorders and paraneoplastic syndromes are very difficult to do, especially in patients with atypical clinicopathologic features, such as in the present case. In more than 50% of cases, the SCLC cells secrete anti-Hu antibodies, and patients present paraneoplastic encephalomyelitis and/or peripheral neuropathy.^1^ However, as long as 40% of patients with paraneoplastic encephalomyelitis are seronegative,^1,4^ these onconeural antibodies do not have real value in clinical practice.

The occurrence of paraneoplastic retinopathy without associated neuritis increases the difficulty of understanding the pathomechanism of SCLC-associated polyneuropathy.^10^ Some suppositions have been put forward, such as autoimmunity between tumor cells and host tissues.^10^

Regarding paraneoplastic Guillain-Barre syndrome, this has been reported in fewer than 80 PubMed-cited papers as the first clinical presentation of less than 1% of malignant tumors.^4,8,11^ The first report in the field was published by Rohmer et al (1962)^12^ in a patient with solitary myeloma. Up until then, this syndrome had been noted as the first clinical sign in patients with Hodgkin lymphomas and carcinomas, independently of their location or histologic type and in most cases having a subacute evolution.^8,11^ The exact mechanism of Guillain-Barre syndrome is not known; some authors consider its association with cancer to be incidental, whereas others argue, based on the increased serum level of IgG autoantibodies against the GM1 ganglioside and of anti-Hu and onconeural antibodies against antigen-specific T lymphocytes, for a severe cross-immunoreaction directed at the tumor and peripheral nerves and a subsequent progressive inflammatory loss of dorsal root ganglia.^4,5,8,11,13^ However, our patient had a fatal axonal form of Guillain-Barre-like syndrome, the axonal degeneration in the nerve roots and distal peripheral nerves being probably secondary installed as a result of acute disruption of the anterior horn and dorsal root ganglia by the tumor cells.^6,9^ Loss of deep tendon reflexes could be explained by the possible autoimmune demyelinating polyneuropathy.^5^

Differential diagnosis of paraneoplastic Guillain-Barre syndrome should take into account other nonneoplastic lesions, such as autoimmune diseases, diabetes mellitus, and alcoholic neuropathy. In lung-associated lesions, a viral polyneuropathy, such as infection with cytomegalovirus or Epstein Barr virus and *Campylobacter jejuni* enteritis,^8,14^ should also be excluded. In the present case, a rapid evolution and axonal predominance, compared with classic subacute Guillain Barre syndrome, was helpful in the correct diagnosis, but the particular “coin-like” peripheral location of the tumor was confusing for the imagistic investigation and even for the macroscopic examination of the lungs at autopsy.

Independent of its pathogenetic mechanism, Guillain-Barre-like syndrome is a rare condition that is very difficult to diagnose and treat. It afflicts 1 in every 100,000 persons each year,^8^ severely affects the quality of life of patients, and, in most cases, leads to death in a short period of time. Thus, in any patient with ascending neuropathy, lung cancer with atypical imagistic features should be taken into account, necessitating supplementary positron-emission tomography-CT examinations in the most difficult cases.
